# Transcriptomic Insights into Salt Stress Response in Two Pepper Species: The Role of MAPK and Plant Hormone Signaling Pathways

**DOI:** 10.3390/ijms25179355

**Published:** 2024-08-29

**Authors:** Muhammad Aamir Farooq, Muhammad Zeeshan Ul Haq, Liping Zhang, Shuhua Wu, Naveed Mushtaq, Hassam Tahir, Zhiwei Wang

**Affiliations:** 1 Key Laboratory for Quality Regulation of Tropical Horticultural Crops of Hainan Province, School of Breeding and Multiplication (Sanya Institute of Breeding and Multiplication), Center of Nanfan and High-Efficiency Tropical Agriculture, Hainan University, Sanya 572025, China; 2 Key Laboratory for Quality Regulation of Tropical Horticultural Crops of Hainan Province, School of Tropical Agriculture and Forestry, Hainan University, Haikou 570228, China

**Keywords:** pepper, salt stress, transcriptome, WGCNA, enzymatic antioxidants

## Abstract

Salt stress imposes significant plant limitations, altering their molecular, physiological, and biochemical functions. Pepper, a valuable herbaceous plant species of the *Solanaceae* family, is particularly susceptible to salt stress. This study aimed to elucidate the physiological and molecular mechanisms that contribute to the development of salt tolerance in two pepper species (*Capsicum baccatum* (moderate salt tolerant) and *Capsicum chinense* (salt sensitive)) through a transcriptome and weighted gene co-expression network analysis (WGCNA) approach to provide detailed insights. A continuous increase in malondialdehyde (MDA) and hydrogen peroxide (H_2_O_2_) levels in *C. chinense* and higher activities of catalase (CAT), superoxide dismutase (SOD), and peroxidase (POD) in *C. baccatum* indicated more tissue damage in *C. chinense* than in *C. baccatum.* In transcriptome analysis, we identified 39 DEGs related to salt stress. Meanwhile, KEGG pathway analysis revealed enrichment of MAPK and hormone signaling pathways, with six DEGs each. Through WGCNA, the ME.red module was identified as positively correlated. Moreover, 10 genes, *A-ARR* (CQW23_24856), *CHIb* (CQW23_04881), *ERF1b* (CQW23_08898), *PP2C* (CQW23_15893), *ABI5* (CQW23_29948), *P450* (CQW23_16085), *Aldedh1* (CQW23_06433), *GDA* (CQW23_12764), *Aldedh2* (CQW23_14182), and *Aldedh3* (CQW23_11481), were validated by qRT-PCR. This study provides valuable insights into the genetic mechanisms underlying salt stress tolerance in pepper. It offers potential targets for future breeding efforts to enhance salt stress resilience in this crop.

## 1. Introduction

Abiotic stresses pose significant limitations on crop yield [1]. These stresses, such as salinity, temperature fluctuations, drought, flooding, and heavy metal, profoundly impact the growth and yield of crops [2,3]. Alarmingly, around 90% of arable lands are susceptible to one or more of these stressors [4], resulting in yield losses of up to 70% in major food crops [5]. Abiotic stress triggers molecular, physiological, and biochemical alterations in plants, leading to changes in morphology and disruptions in plant functions [6,7]. Soil salinity significantly reduces crop yield across various global regions [8]. Currently, cultivated and irrigated lands afflicted by salt constitute about 20–30% of the worldwide soil area, with projections indicating a potential doubling of salinized regions by 2050 [9]. This increase is attributed to high evapotranspiration rates, inadequate rainfall, unsustainable agricultural practices, and ineffective irrigation and drainage methods [10]. Consequently, conventional high-yielding crop varieties often struggle to tolerate these elevated salt concentrations [11].

Salinity triggers complex mechanisms within plants, impacting various physiological, biochemical, and molecular pathways [12]. Initially, it diminishes soil osmotic potential, imposing osmotic stress that reduces root water content and nutrient uptake [13]. Furthermore, salinity stress induces the overproduction of reactive oxygen species (ROS), leading to oxidative damage in cellular components such as the plasma membrane, mitochondria, and chloroplasts [14]. This oxidative stress disrupts protein biosynthesis, ion homeostasis, and photosynthesis while downregulating antioxidant activities within the plant cell [15]. To avoid oxidative stress damage, plants activate antioxidant enzymes like superoxide dismutase (SOD), peroxidase (POD), and catalase (CAT) and accumulate nonenzymatic antioxidant compounds, including primary and secondary metabolites like proline, phenolic acids, carotenoids, or alkaloids [16]. Similarly, ROS initiation during salt stress triggers Mitogen-activated protein (MAP) cascades and hormonal and other metabolic pathways aiding ionic balance and osmotic equilibrium [17]. MAPK responds to osmotic stress by elevating osmolyte synthesis [18]. MAPKs are supposed to maintain ROS balance [19]. Phytohormones, like ABA (abscisic acid), also regulate osmotic stress caused by salinity and ROS-mediated pathways [20]. ABA also influences ion balance, elevating K^+^ and Ca^2+^ and hindering Na^+^ and Cl^−^ absorption [21]. It controls Na^+^/K^+^ ions and H_2_O_2_ concentrations during salt stress [22], activating MAPK via H_2_O_2_ from ROS scavenging [23].

Pepper is an important member of the Solanaceae family, with a broad diversity in fruit morphology and flavor [24]. It originated from Central/South America and Mexico and later spread globally to Europe, Asia, and Africa. Its production has increased over the last 20 years from 17 to 36 million tons, and the cultivated area has expanded by about 35% worldwide [25]. It is cultivated in most regions of China, particularly in the east, west, and south, with a share of 7.76% of the total national vegetable output [26]. It is a tropical and subtropical annual crop that requires a warm (18–30 °C) and humid climate for optimal growth and fruit production [27]. Still, it can also be cultivated in temperate climates in greenhouses [28]. Several stresses (biotic and abiotic) significantly affect peppers, which decrease yields and fruit quality [29]. In the previous study, salt stress is considered most relevant for pepper plants and has moderately sensitive, sensitive, or highly susceptible effects [30]. A high salt concentration source that affects plants may be soil or irrigation water [31]. In pepper plants, the dry weight and marketable yield diminish by 46% and 25%, respectively, when rinsed with water at 4.4 dS m^−1^ [32].

Previous research has delved into salt-related genes and pathways in model plants, overlooking pepper [33]. Pepper is particularly susceptible to salt stress, so it is essential to understand the molecular mechanisms governing its response to this environmental challenge. We examined two pepper species, *Capsicum baccatum* (moderately sensitive) and *Capsicum chinense* (sensitive), in response to salt stress. This study aims to compare the physiological responses, identify enriched pathways and DEGs, and identify potential genetic targets. These findings will contribute to a deeper understanding of pepper’s intricate molecular mechanisms underlying salt stress tolerance. It offers potential targets for future breeding efforts to enhance salt stress resilience in this economically important crop species.

## 2. Results

### 2.1. Physiological and Phenotypical Alterations

**Figure 1:**
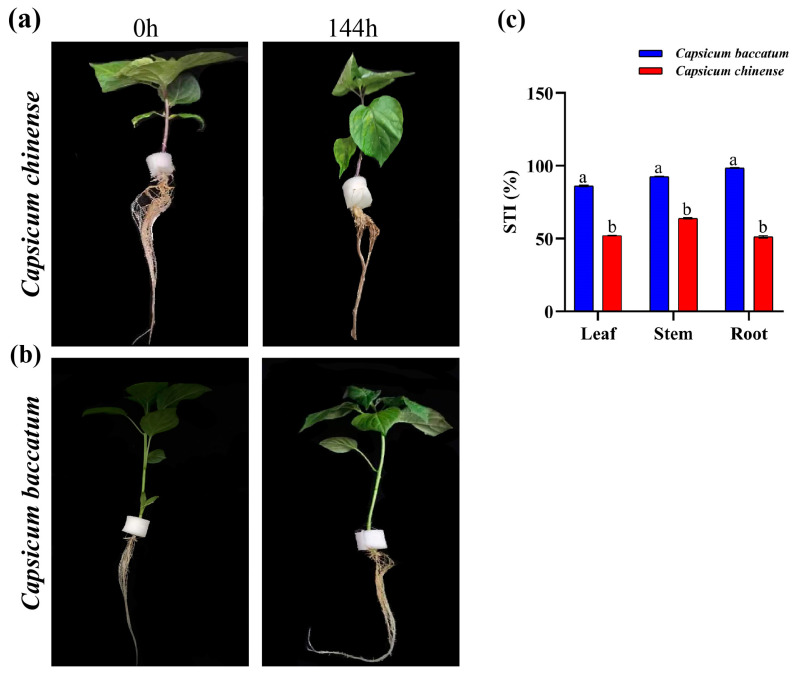
(**a**) The external appearance of the leaves and roots from *C. chinense* exposed to salt (NaCl) stress was observed at various time intervals (0 h and 144 h). (**b**) The external appearance of the leaves and roots from *C. baccatum* exposed to NaCl stress was observed at various time intervals (0 h and 144 h); (**c**) stress tolerance index percentage (STI %) of leaf, stem, and root dried samples of *C. baccatum* and *C. chinense* at 100 mM NaCl stress. The lowercase alphabets indicating significant differences at *p* < 0.05.

Following 24 h of exposure to salt stress, observable alterations began to occur in the *C. chinense* plants, with the lower leaves exhibiting a gradual yellowing and a decline in turgor pressure compared to the *C. baccatum* plants, which became more pronounced by the 144 h mark (Figure 1).

**Figure 2:**
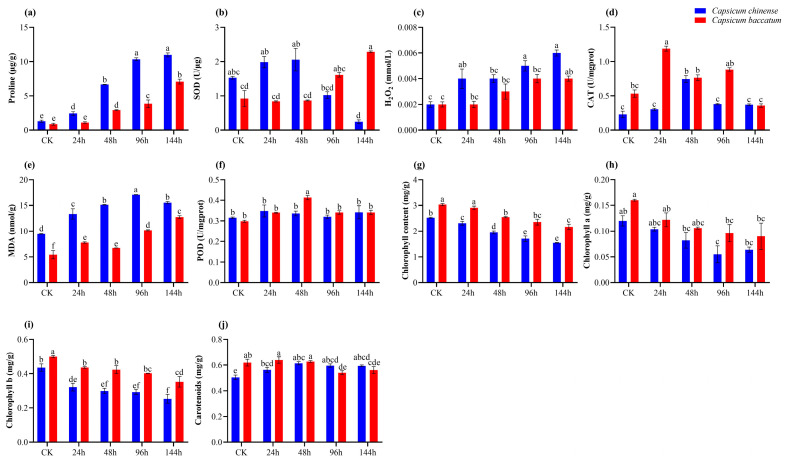
The biochemical parameters are (**a**) Proline, (**b**) SOD, (**c**) H_2_O_2_, (**d**) Catalase, (**e**) MDA, (**f**) POD, (**g**) Chlorophyll contents, (**h**) Chl. a and (**i**) Chl. b, and (**j**) Carotenoids of *C. chinense* and *C. baccatum* leaves under salt stress at various intervals. The presented data is the average ± standard deviation of three replicates, with lowercase alphabets indicating significant differences at *p* < 0.05.

Proline levels exhibited a more significant increase in *C. chinense* compared to *C. baccatum* at all time points. The highest proline level was observed in *C. chinense* at 144 h (Figure 2a). In *C. chinense*, the highest level of SOD was observed at 24 h and 48 h compared to its control (CK; 0 h). However, in *C. baccatum*, there was an initial decrease in SOD levels at 24 h and 48 h compared to its respective CK. Interestingly, in *C. baccatum*, the SOD levels peaked at the 144 h time point compared to CK, whereas in *C. chinense*, a decrease in SOD levels was observed at 144 h when compared to the other time points and CK (Figure 2b). The highest H_2_O_2_ level was documented at 144 h in *C. chinense* in contrast to *C. baccatum*, which remained consistently higher at all time points and compared to CK. Conversely, the maximum H_2_O_2_ activity in *C. baccatum* was recorded after 24 h of salt stress across all time points and compared to CK (Figure 2c). Similarly, CAT activity peaked after 24 h of stress in *C. baccatum* and compared to CK, while it displayed a declining trend, particularly pronounced in *C. chinense* over time and compared to CK (Figure 2d). Nonetheless, the peak level of MDA was documented in *C. chinense* at 96 h compared to CK, surpassing that of *C. baccatum* at all time points and CK (Figure 2e). Conversely, POD activity exhibited its highest levels in *C. baccatum* compared to *C. chinense*, particularly evident after 48 h of salt stress when compared across all time points and compared to CK (Figure 2f). Furthermore, compared to the CK of each species, it was apparent that total chlorophyll contents, Chl a and Chl b contents, exhibited a more pronounced decrease in *C. chinense* than in *C. baccatum* (Figure 2g–i). In contrast, carotenoid levels in *C. baccatum* showed an increase up to 24 h and 48 h after salt stress, surpassing the levels in *C. chinense* at these time points, but displayed a more substantial decrease in carotenoids in *C. baccatum* than in *C. chinense* at the 144 h mark following salt stress (Figure 2j). Overall, the physiological indicators suggest that *C. chinense* exhibits a higher sensitivity to salt stress when compared to *C. baccatum*.

### 2.2. Stress Tolerance Index Percentage (STI%)

The STI% results showed that the *C. baccatum* root showed more salt stress tolerance than the stem and roots as compared to the *C. chinense* root, stem, and leaf (Figure 1c). The STI% showed *C. baccatum* tolerance against salt stress. 

### 2.3. Microscopic Scanning of Leaf and Root Tissues

**Figure 3:**
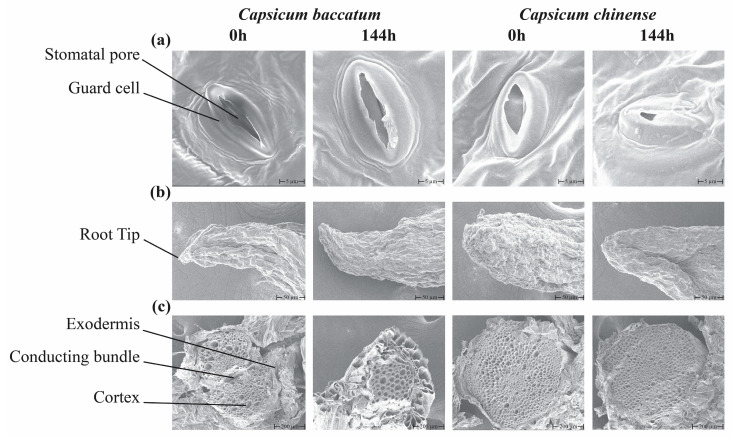
The SEM images of the leaf stomata (**a**), root tips (**b**), and root cross-section (**c**) of *C. baccatum* and *C. chinense* exposed to salt stress were observed at 0 h and 144 h.

We evaluated the leaf stomata, root structure, and cross-section in both species treated with salt stress (Figure 3). SEM results showed that compared to their respective controls, *C. chinense* salt-treated samples stomata showed more closing as compared to *C. baccatum*; the root scan and its cross-section analysis also showed weak health due to the change in the root tip appearance, degradation of vascular tissues, and increased cell wall thickening in the cortex region as compared to *C. chinense* control and *C. baccatum* control and treated samples (Figure 3a,b). Moreover, a noticeable decline in leaf stomata and cross-section in length, width, pore length, and pore width of *C. chinense* was recorded compared to its control and *C. baccatum* control and treatment samples (Supplementary Table S1). These results showed *C. chinense* sensitivity against salt stress. 

### 2.4. Analysis of RNA Sequencing

**Figure 4:**
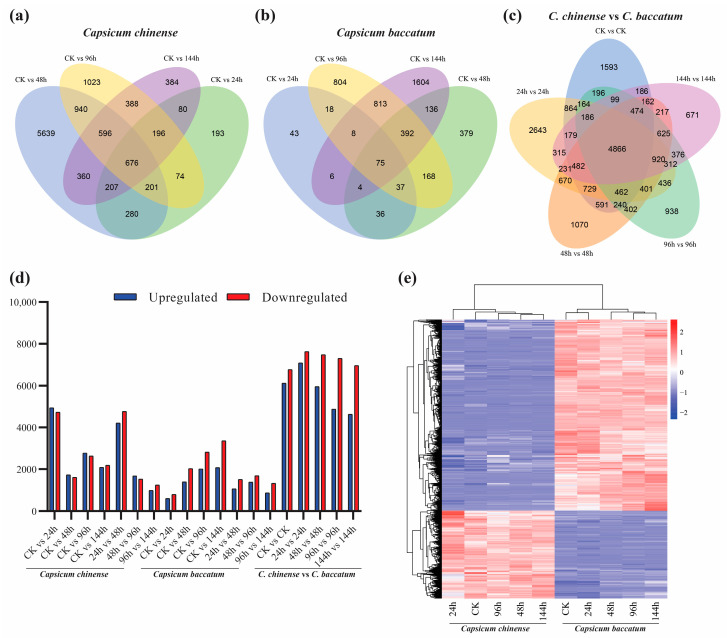
Venn diagrams were constructed to represent commonly expressed genes in different comparisons: (**a**) all treatments paired with control in *C. chinensis*, (**b**) all treatments paired with control in *C. baccatum*, and (**c**) amongst and between control pairs and treatment pairs of both *C. chinensis* and *C. baccatum*, under salt stress. Meanwhile, (**d**) shows the overall number of upregulated and downregulated genes within and between the species at all-time points in salt stress response; moreover, the gene heat map (**e**) shows their respective pattern under salt stress.

Transcriptome sequencing of pepper cultivars *C. chinense* and *C. baccatum* yielded valuable and distinct data about their responses under salt stress conditions. The essential RNA-seq characteristics are summarized in Supplementary Table S2. Upon scrutinizing the raw sequencing data, it was determined that *C. chinense* generated a range of 38–56 million clean reads, while *C. baccatum* produced 38–45 million clean reads. The data exhibited a consistent overall error rate of 0.03%, with quality scores (Q20 ≤ 95–97% and Q30 ≤ 92–94%) and a GC content ranging from 40% to 43%, as summarized in Supplementary Table S2. Among all the different comparisons, 33,022 genes were detected across and within both species. Moreover, we constructed Venn diagrams based on the control with all-time points within the species and between the species; 675 genes were detected among the controls and all-time points in *C. chinense* (Figure 4a), 75 in *C. baccatum* (Figure 4b), and 4866 genes among control groups of both species with all-time points group (Figure 4c), respectively. Moreover, upregulated and downregulated genes were shown among all-time points within and between species (Figure 4d), where maximum upregulated and downregulated genes were detected after 24 h in the salt stress group of both species. Similarly, the heat map of the genes is given in Figure 4e.

### 2.5. Identification of the Module Using WGCNA

**Figure 5:**
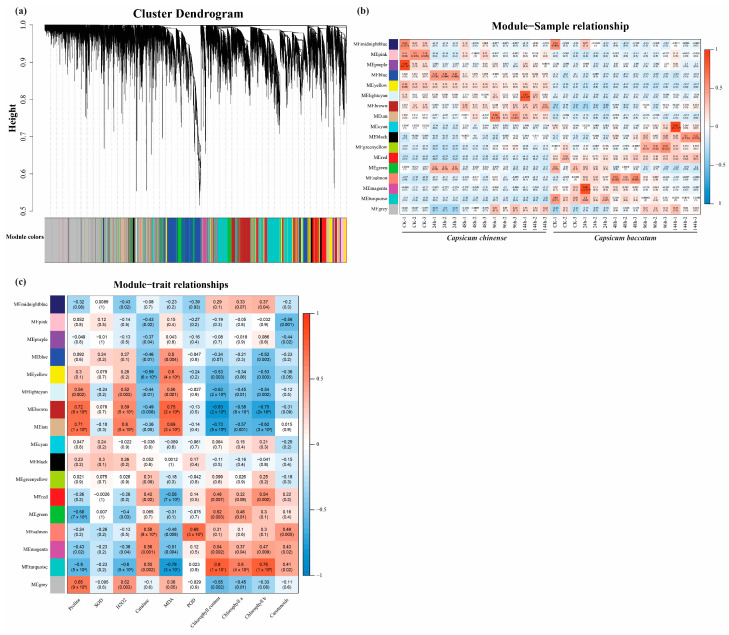
(**a**) The genes have been classified into 17 discrete modules, as seen in the sequence diagram in the figure’s uppermost section. The lower portion of the diagram depicts the gene modules using a range of colors, along with the corresponding count of bases (**b**,**c**). The abscissa is the sample, the ordinate is the module, and the number of each grid represents the correlation between the module and the sample. The closer the value is to 1, the stronger the positive correlation between the module and the sample. The closer it is to −1, the stronger the negative correlation between the module and the sample. The number in parentheses represents the *p*-value of significance, and the lower the number, the stronger the significance (denoted as **b**,**c**). However, Figure (**c**) depicts the physiological traits of the abscissa. The presented heat map visually represents the eigengene adjacency for each module, indicating the degree of correlation between these modules. The coefficient correlation between the gene module and the sample/trait is determined within each cell, and subsequently, the corresponding *p*-value is presented directly below.

To evaluate the expression patterns of pepper genes linked to salt stress, we developed a gene co-expression network using the WGCNA method. A comprehensive set of 16,117 genes was classified into 17 co-expression modules, with each module being visually distinguished by a unique shade. These modules were subsequently utilized for further research, as seen in Figure 5a. Significantly, our study revealed the presence of two modules strongly associated with salt resistance, as evidenced by a *p*-value ≤ 0.05. The ME.red module had a favorable correlation with resistance to salt stress (Figure 5b), including transcription factors like *ERF1b2*, *A-RR9*, ABA receptor *PYL4/6*, *P450*, *pKinase*, low kDa *HSP*, etc. Conversely, the ME.yellow module displayed a favorable association with physicochemical properties (Figure 5c). On initial findings, we identified 1059 genes within the ME.red module, and pathway enrichment analysis of the genes within the ME.red module revealed that the primary enriched pathways were related to plant MAPK and hormone signal transduction.

### 2.6. GO and KEGG Enrichment Analysis

**Figure 6:**
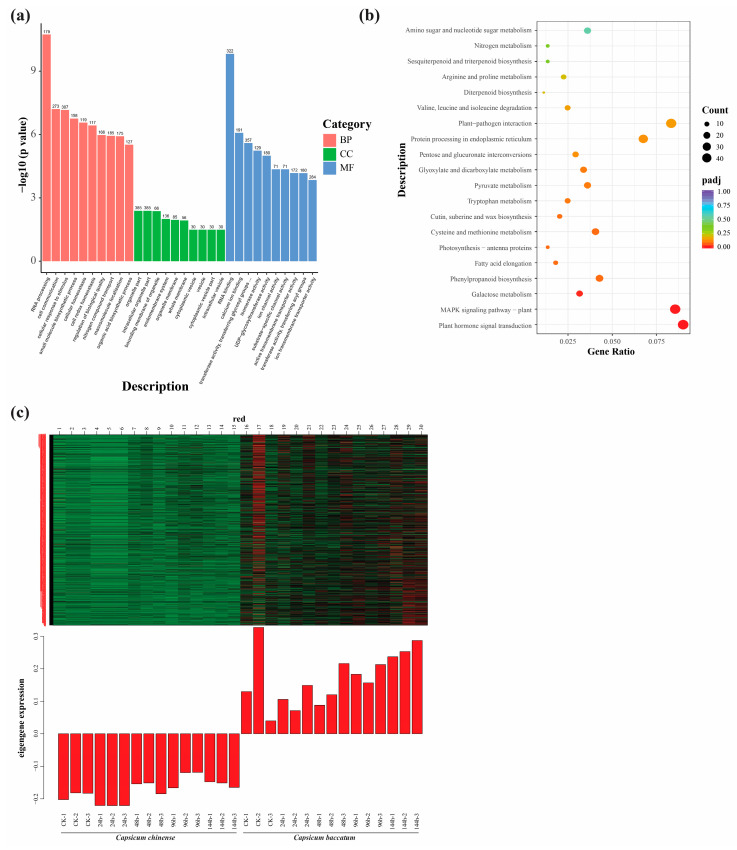
GO (**a**) and KEGG (**b**) enriched DEGs functional analyses; (**c**) expression pattern of genes in the ME.red module selecting using the log10 FPKM values.

Enrichment analyses like GO and KEGG were applied to find significant pathways under 100 mM salt stress. We employed GO functional enrichment analysis to categorize the functions of the identified DEGs in response to salt stress. For Biological Process (BP), the most prominent processes included RNA processing (GO:0006396), cell communication (GO:0007154), and cellular response to stimulus (GO:0051716) processes, featuring 179, 273, and 307 genes, respectively. Similarly, for Cellular Components (CC), the top three enriched components were the organelle part (GO:0044422), intracellular organelle part (GO:0044446), and bounding membrane of organelle (GO:0098588), with 385, 385, and 66 genes. However, for the Molecular Function (MF), the three most important categories were RNA binding (GO:0003723), calcium ion binding (GO:0005509), and transferase activity, transferring glycosyl groups (GO:0016757) with 322, 191, and 357 genes, respectively (Figure 6a).

KEGG pathway enrichment analysis was performed for both cultivars exposed to salt stress. Among the top 20 pathways, the plants MAPK signaling (cann04016) and plant hormone signal transduction (cann04075) pathways, phenylpropanoid biosynthesis (cann00940), galactose metabolism (cann00052), protein processing in endoplasmic reticulum (cann04141), and cysteine and methionine metabolism (cann00270) pathways were mainly enriched pathways (Figure 6b). Hence, considering the outcomes from the KEGG analysis, the plant MAPK signaling and hormone signal transduction pathways were singled out as the most crucial pathways, with pivotal roles in regulating the salt stress response in pepper. The DEGs of these pathways were also positively associated with the ME.red module in WGCNA. Moreover, the epigene expression heat map of the ME.red module is given below (Figure 6c). 

### 2.7. MAPK Signaling Pathway

**Figure 7:**
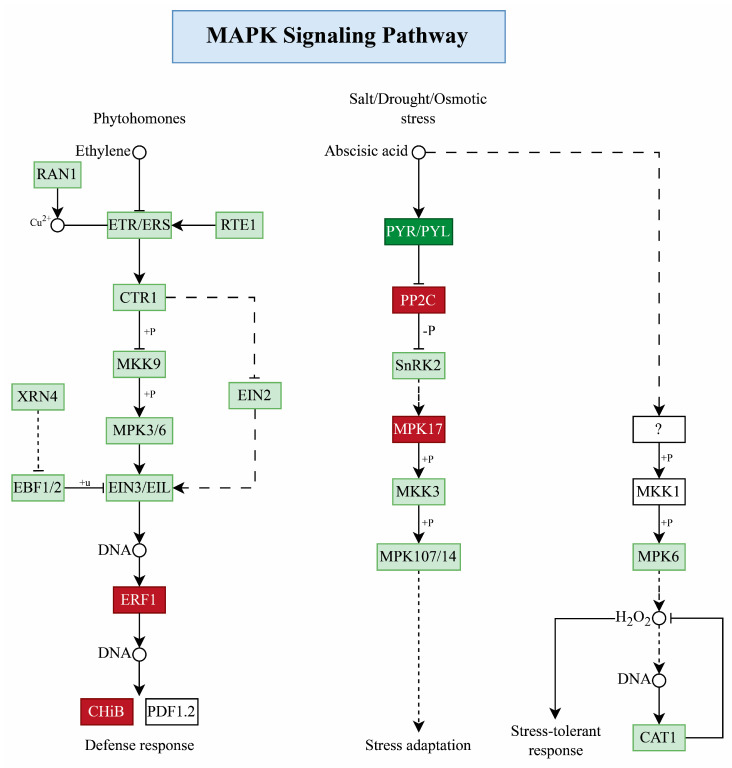
Plant MAPK signaling pathway; upregulated genes are represented in dark red, while the downregulated gene is expressed in dark green color.

This work identified six DEGs highly enriched in the MAPK signaling pathway in pepper. These DEGs are known to have a role in the defensive response and adaptation to various abiotic stimuli, including salt, drought, and cold stress. The dynamic transcriptome analysis showed that *ERF1b* transcription factor (TF) upregulation in both species interacts with acidic endochitinase *CHIb* in the defense response of the phytohormone ethylene pathway. However, *ERFb* showed more expression in *C. baccatum* than in *C. chinense*, while acidic *CHIb* showed more expression in *C. chinense*, indicating a possible role in the pepper response to salinity (Figure 7). Similarly, in stress adaptation, we found four TFs, where an abscisic acid receptor *PYL4* downregulation inhibits two *PP2C* (protein phosphatase 2C 51 and probable protein phosphatase 2C 24) that interact with the *MAPKKK18*. However, both *PP2C* TFs showed upregulation in both species cultivars, but more upregulation was seen in *C. chinense* than *C. baccatum*, while *MAPKK18* showed more expression in *C. baccatum* than *C. chinense*, which shows their probable role in the pepper response to salt stress (Figure 7). 

### 2.8. Plant Hormone Signal Transduction Pathway

**Figure 8:**
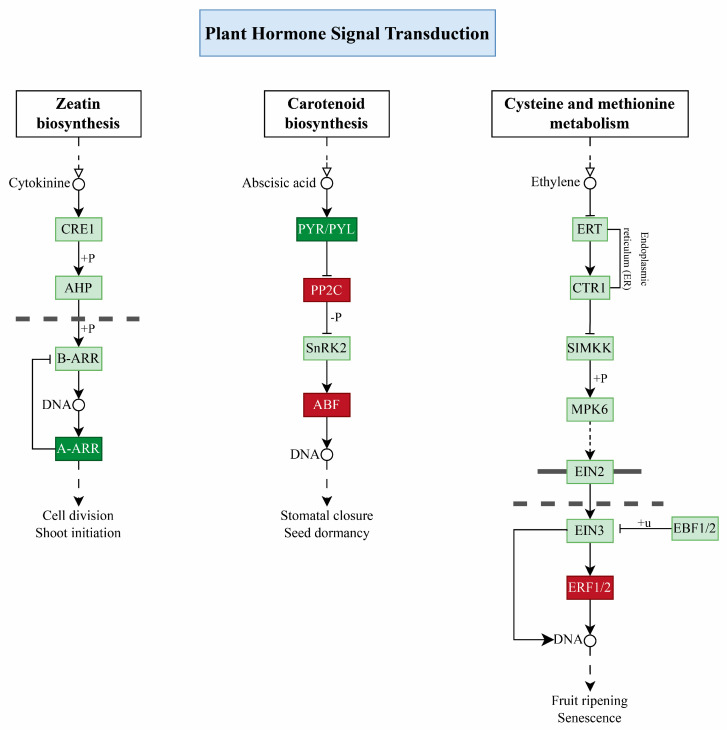
In the Plant hormone signal transduction pathway; upregulated genes are represented in dark red, while the downregulated genes are expressed in dark green.

Under salt stress, cytokinins (Ck) are involved in cell division and shoot initiation during the zeatin biosynthesis process. In this process, Ck negative regulator *A-AAR* downregulation irreversibly inhibits *B-ARR* in *C. chinense* while it was absent in *C. baccatum*, which shows it might have a role in sensitivity against salinity. Similarly, in carotenoid synthesis under plant hormonal signal transduction, ABA plays a crucial role in stomatal closure and seed dormancy. In this process, ABA receptor *PYL4* downregulation inhibits *PP2C* (protein phosphatase 2C 51 and probable protein phosphatase 2C 24), which upregulates and interacts with *ABF* TF, *BZIP_1* (Abscisic acid–insensitive 5-like protein 5). Interestingly. *ABI5* was found to be downregulated in *C. chinense* and upregulated in *C. baccatum*, indicating its possible role in pepper sensitivity against salinity stress (Figure 8). Moreover, in cysteine and methionine metabolism, ethylene, as an important phytohormone, functions as fruit ripening and senescence under stress conditions; in our study, *ERF1b* was found to be upregulated more in *C. baccatum* than *C. chinense*, which predicts it may have a role in pepper response towards salt stress. 

### 2.9. qRT-PCR Validation

**Figure 9:**
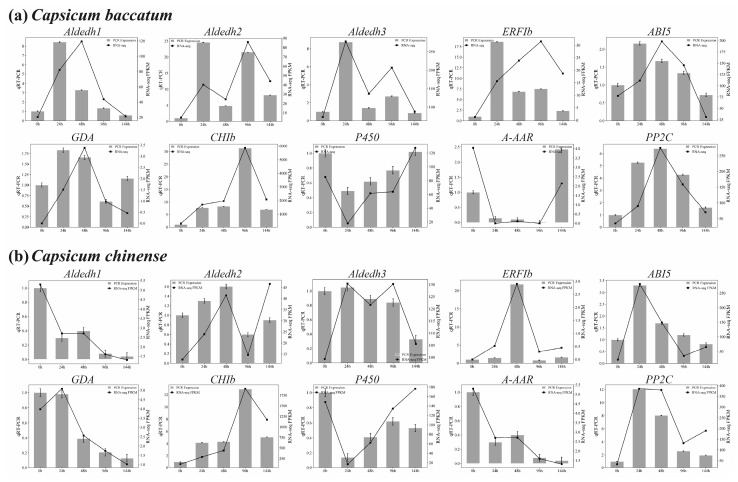
RNA-Seq transcriptional data validation of randomly selected DEGs through qRT-PCR between *C. baccatum* (**a**) and *C. chinense* (**b**) against salt stress; represents the same DEGs comparison between these two species.

The qRT-PCR analysis validated RNA-seq expression patterns of 10 specifically selected DEGs. This study was performed across five specific time periods when salt stress treatment was applied in both *C. baccatum* and *C. chinense*, as depicted in Figure 9. Consistent outcomes were reported across multiple genes, encompassing those implicated in MAPK and plant hormone signal transduction pathways (*A-ARR*, *CHIb*, *ERF1b*, *PP2C*, and *ABI5*), stilbenoid, diarylheptanoid, and gingerol biosynthesis (*P450*), alanine, aspartate, and glutamate metabolism (*Aldedh1*), phenylpropanoid biosynthesis (*GDA*), and arginine and proline metabolism (*Aldedh2* and *Aldedh3*) (Figure 9a,b). The expression patterns obtained from RNA-Seq data and quantitative real-time polymerase chain reaction (qRT-PCR) demonstrated a significantly strong positive correlation across various time points.

## 3. Discussion

### 3.1. Phenotypical and Physiological Changes

Salinity poses a significant challenge to plant growth and development, affecting crucial processes such as water absorption [34]. This environmental stressor alters plants’ biochemical, physiological, and morphological reactions, ultimately reducing growth, yield, and quality. To cope with salt stress, plants have evolved mechanisms to manage water relations and accumulate ions, thereby developing tolerance to osmotic stress through the accumulation of organic osmolytes or ions [35]. Salt-tolerant plants also regulate their levels of sodium (Na^+^) and chloride (Cl^−^) ions by transporting or storing them in vacuoles to prevent their toxicity [36]. A high salt level triggers the production of ROS, which can cause damage to proteins, lipids, and DNA [37]. To respond to ROS, plants activate their defense system, which includes POD, SOD, CAT, and other enzymatic antioxidant enzymes and some nonenzymatic compounds like proline, carotenoids, and other such compounds [38]. These enzymatic antioxidants and compounds are crucial for cell growth and survival to scavenge ROS in maintaining the ROS and antioxidant defense balance [39]. In the present study, under salt stress, we investigated the response of two pepper species, *C. baccatum* and *C. chinense*. We observed a distinct pattern of enzymatic and no enzymatic activities in both species. At 24 h and 48 h after salt stress treatment, *C. chinense* showed an early and sustained increase in SOD activity, as compared to *C. baccatum* with a delayed response initially followed by a peak at 144 h. The CAT activity peaked after 24 h in *C. baccatum*, whereas it continuously declined over time in *C. chinense*. Similarly, POD activity was higher in *C. baccatum* compared to *C. chinense* in a continuous manner, particularly after 48 h of treatment. These outcomes highlight the importance of enzymatic antioxidant activities in scavenging the ROS to avoid membrane damage [40], which indicates a plant’s response to stress defense and resistance [41].

Moreover, our investigation also revealed the proline, H_2_O_2_, and MDA role between both species under salt stress. We noticed a greater increase in proline levels in *C. chinense* at all time points compared to *C. baccatum.* Similarly, H_2_O_2_ remained consistent in *C. baccatum*, but it peaked at 144 h in *C. chinense*, at all-time points showing consistency with earlier findings that high salt stress can cause H_2_O_2_ rapid bursts in plant cells, which could be an important signal for salinity response [42]. We recorded a maximum level of MDA in *C. chinense* at 96 h after salt stress compared to *C. baccatum* at all time intervals, which suggests greater membrane damage in salt-sensitive species, consistent with the earlier research [43,44]. Additionally, this study also found a more consistent decline in chlorophyll in *C. chineses* under salt stress as compared to *C. baccatum*, and these findings are in agreement with studies showing that salt-tolerant plants normally experience less chlorophyll decrease or degradation under abiotic stresses [45,46,47].

### 3.2. Role of MAPK Signaling Pathway

Salinity adversely affects the crop plants’ biochemical and physiological pathways [48]. We investigated the molecular response of *C. baccatum* and *C. chinense* under NaCl stress, focusing on the MAPK signaling pathway. Under salt stress, the ethylene-responsive factor (*ERF1b*) showed upregulation in both species but with more expression in *C. baccatum* and more interaction with *CHIb*, which can activate defense-related genes like *PDF1.2*, potentially indicating its robust defense response as compared to *C. chinense*, consistent with the earlier findings [49]. Similarly, we found that *PYL4* (abscisic acid receptor) downregulation and inhibition of *PP2C* genes (protein phosphate 51C and 24C), which interact with mitogen-activated protein kinases (*MAPKKK18*). For *PP2C*, both genes showed more expression in *C. chinense*, whereas *MAPKKK18* showed more expression in *C. baccatum*. These findings are consistent with prior studies suggesting that plants can adapt to stress conditions through ABA signaling, which involves ABA receptors, pyrabactin-resistant regulatory components (*PYR1*/*PYL*/*RCAR* proteins), and *PP2C* genes [50,51,52]. MAPK cascades are also implicated in ABA signaling processes, which include stomatal regulation and antioxidant defense [53]. For example, in stressful conditions, ABA regulates MAPK signaling components [54]. 

### 3.3. Role Hormone Signal Transduction Pathway

Phytohormones are important players and play a key role in plant growth and development by involving various plant defense mechanisms under salt stress [55]. For instance, ABA, gibberellic acid (GA), and jasmonic acid (JA) improve alfalfa’s salt stress response [56]. Similarly, in regulating the effects of salinity-related factors, ethylene works with a range of other phytohormones and stress signaling molecules to finely orchestrate the plant’s responses, whether in the context of salinity or normal growth [57,58]. Our study delves into the key regulatory roles of phytohormones like Cks, ABA, and ethylene in shaping pepper crop transcriptional mechanisms under salt stress. Cks are important indicators of plant sensitivity to salinity, as they influence different physiological processes [52,59]. In our study, we observed the downregulation of *ARR 4/9* signaling genes in *C. chinense*, which might negatively impact the Ck pathway, indicating their potential involvement in salt response; these results are consistent with prior findings [60,61]. Previous research suggests ethylene is a significant regulator of NaCl stress response at various cellular levels and transcription factors [62]. Our findings revealed a higher upregulation of *ERF1b* expression in *C. baccatum* than in *C. chinense* under stress conditions, indicating their importance in pepper’s tolerance response. Moreover, ABA’s vital role in salt stress responses indicates its influence on various physiological mechanisms [63]. In our study, the downregulation of the ABA receptor *PYL4* inhibits *PP2C* genes, leading to the upregulation of *bZIP_1* (ABI5), which positively impacts the ABA signaling pathway in pepper under salt stress. *ABI5* showed a higher expression in *C. baccatum* than in *C.chinense*, which indicates that *ABI5* might have a role in tolerance against salt stress. Prior findings support our findings by emphasizing the significance of ABA, its receptors, *PP2C* phosphatases, and ABF transcription factors in plant responses under salt stress [64,65]. This study underscores the importance of the intricate interplay of phytohormone signaling pathways in regulating pepper’s response to salt stress, providing valuable insights into potential targets for improving salt tolerance in crops. Further research must elucidate the specific mechanisms underlying hormone-mediated responses to salt stress in pepper plants.

## 4. Materials and Methods

### 4.1. Plant Material and NaCl Treatments

Two pepper-cultivated species seeds were germinated on MS media and screened against 100 mM salt stress. The *C. baccatum* germplasm (HNUCB226), moderately salt-sensitive, and *C. chinense* germplasm (HNUCC275), salt-sensitive (based on screening), were selected for this study [66]. At the 3–4 leaf growth stage (BBCH 13–14), seedlings were transferred into 800 mL boxes filled with Hoagland nutritional solution and grown in a growth chamber (PQX-330B-30HM, Ningbo Life Technology Co. Ltd., Ningbo, China) under conditions of 26 °C temperature, 70% humidity, and a light cycle of 16 h followed by 8 h of darkness. After 4–5 weeks of growth, the plants were treated with 100 mM NaCl for 144 h [67]. A total of 72 plants were selected, with 36 plants in each species; each species contained 3 replicates, and each replicate contained 12 plants. After stress treatment, samples were collected at 0 h, 24 h, 48 h, 96 h, and 144 h, respectively, with three biological repeats. Subsequently, the collected samples were stored at −80 °C for future analysis.

### 4.2. Response of Biochemical Indexes

To assess the enzyme (SOD, POD, and CAT) activities, 0.1 g of leaf samples were homogenized in 900 µL of phosphate buffer (pH 7.8). Samples were centrifuged (TGL-1850 Micro refrigerated centrifuge, Sichuan Shuke Instrument Co., Ltd., Chengdu, China) at 10,000× *g* for 10 min, and the supernatants were used to determine the antioxidant enzyme activities according to their respective kits (A001-1-2, A084-3-1, and A007-1-1) from Nanjing Jiancheng Biotechnology. Additionally, proline and MDA content and H_2_O_2_ concentration content in pepper leaves were quantified using assay kits (G0111F96, G0110F, and A064-1-1) from Suzhou Geruisi Biotechnology Co. Ltd., (Suzhou, China) and Nanjing Jiancheng Biotechnology (Nanjing, China), adhering to the instructions provided by the manufacturers, respectively. Moreover, for the determination of the total chlorophyll contents, chlorophyll a, chlorophyll b, and carotenoids, 0.1 g of fresh leaf samples were ground with 10 mL of extraction solution. Samples were centrifuged at 10,000× *g* for 10 min, and the supernatant was used to observe the values of Chl a, Chl b, and carotenoid according to the commercially available kit (G0163F) from Suzhou Geruisi Biotechnology Co. Ltd., Suzhou, China.

### 4.3. Stress Tolerance Index Percentage (STI%)

The leaf, stem, and root samples from the control and treatment of both species were dried in an oven (DGG-9053A, Shanghai Enxin Instrument Co., Ltd., Shanghai, China) at 65 ℃ for 72 h. Samples were weighed via a weighing apparatus, and stress tolerance index percentage (STI%) was calculated using the following formula previously described [68].

STI (%) = (Dry weight of leaf/stem/root of treated samples/Dry weight of leaf/stem/root of control samples) × 100.

### 4.4. Scanning of Leaf and Root

Root and leaf samples were washed with 75% ethanol and were subsequently dried. After drying, samples were attached to metallic stubs with scotch tape and sputter coated with gold and aluminum in a J20 Ion Sputter Coater for 90 s before scanning. Finally, images were taken using a scanning electron microscope (Thermoscientific Verios G4 UC) [69].

### 4.5. RNA Extraction, Library Construction, and RNA Sequencing

RNA extraction was performed on all obtained samples using the NEB extraction kit. In a subsequent step, the cDNA library was constructed using the RNA Library Prep kit for Illumina^®^ (NEB, Ipswich, Massachusetts, USA). Clusters were generated, and sequencing was performed using the Illumina Novaseq platform, producing paired-end reads with a length of 150 base pairs. The HISAT2 alignment tool mapped paired-end clean reads of both species with the reference genome *C. baccatum* (https://ftp.ncbi.nlm.nih.gov/genomes/all/GCA/002/271/885/GCA_002271885.2_ASM227188v2/GCA_002271885.2_ASM227188v2_genomic.gtf.gz: accessed on 15 May 2023). FeatureCounts (v1.5.0-p3) was used to count the reads numbers mapped to each gene, then the FPKM of each gene was calculated based on the length of the gene and read counts mapped to this gene. Differential expression analysis (DEA) was conducted with DESeq2 software (version 1.20.0). DESeq2 provides statistical routines for determining differential expression in digital gene expression data using a model based on the negative binomial distribution. The resulting *p*-values were adjusted using Benjamini and Hochberg’s approach to control the false discovery rate. Genes with an adjusted *p* ≤ 0.05 found by DESeq2 were assigned as differentially expressed [70]. The clusterProfiler R package (3.8.1) was used to analyze the Gene Ontology (GO) of differentially expressed genes and the statistical enrichment of DEGs in the KEGG pathway [71,72].

### 4.6. Co-Expression Modules Identification

The R WGCNA package (a set of functions used to calculate various weighted association analyses, which can be used for network construction, gene screening, gene cluster identification, topological feature calculation, data simulation, and visualization) was employed to establish a gene co-expression network. The networks were visualized using Cytoscape version 3.6.048. Using the heat map function in the R package, high-throughput heat maps were generated and visually displayed to showcase the gene expression levels for specific DEGs [73].

### 4.7. qRT-PCR Validation

The RNA-seq samples created the quantitative real-time PCR analysis library. Ten randomly selected DEG expression patterns were validated via qRT-PCR. Primers for these genes were designed using PRIMER5 software ((v5.0) Supplementary Table S3). The HiScript III (+gDNA wiper) kit was used for reverse transcription. SYBR qPCR-Mix was used for qPCR analysis, and QuantStudio™ Design and Analysis Software 1.3 calculated the expression levels of each sample. Moreover, the 2^-ΔΔCT^ method was then used to determine the relative expression levels of the chosen DEGs.

### 4.8. Statistics

Data were analyzed using the statistical software SAS 9.4 (SAS Institute Inc., Cary, NC, USA). A two-way ANOVA was employed to analyze the salt stress changes at different intervals. The graphs were generated using GraphPad Prism 8.0.1, and the significant differences were examined using Tukey’s HSD tests following *p* ≤ 0.05.

## 5. Conclusions

This study investigated the physiological and transcriptomic responses of two pepper species (*C. chinense* and *C. baccatum*) to salt stress. Our findings illuminate the complex mechanisms, particularly emphasizing the roles of MAPK and hormone signal transduction pathways. Physiologically, significant differences between the two species were observed, with *C. chinense* exhibiting higher sensitivity, as evidenced by pronounced alterations such as enzymatic antioxidants and nonenzymatic compounds, whereas *C. baccatum* responded more resilient to salt stress. The DEGs involved in plant signaling pathways, including ethylene-responsive factors (*ERFs*), ABA receptors (*PYL*), protein phosphatases (*PP2C*), and basic leucine zipper protein (*BZIP_1*), further highlighted the species-specific responses to salt stress. Overall, this study provides valuable insights into salt stress tolerance’s molecular and physiological mechanisms in pepper plants, identifying key genes and pathways for potential use in breeding salt-tolerant cultivars. These findings also enhance the understanding of plant stress responses, with implications for improving crop resilience to environmental challenges.

## Data Availability

The original data can be found in NCBI BioProject: PRJNA1056267 (https://dataview.ncbi.nlm.nih.gov/object/PRJNA1056267?reviewer=4jvkg624h1schnmnovkjinudoq: accessed on 25 December 2023).

## Supplementary Materials

The following supporting information can be downloaded at: https://www.mdpi.com/article/10.3390/ijms25179355/s1.
